# Clinical efficacy and imaging analysis of oblique lateral lumbar interbody fusion in the treatment of different types of lumbar intervertebral foramen stenosis

**DOI:** 10.1186/s13018-024-04636-9

**Published:** 2024-04-02

**Authors:** Yuan Gao, Fengyu Liu, Zhenfang Gu, Zhengqi Zhao, Yanbing Liu, Kuan Lu, Xianze Sun

**Affiliations:** Department of Spine Surgery, The Third Hospital of Shijiazhuang,, No. 15 Tiyu Street, Shijiazhuang, 050000 China

**Keywords:** Oblique lateral interbody fusion, Lumbar intervertebral foramen stenosis, Nerve decompression, Imaging assessments

## Abstract

**Purpose:**

To analyze and study the clinical efficacy and imaging indexes of oblique lateral lumbar interbody fusion (OLIF) in the treatment of lumbar intervertebral foramen stenosis(LFS) caused by different causes.

**Method:**

33 patients with LFS treated with OLIF from January 2018 to May 2022 were reviewed. Oswestry Dysfunction Index (ODI) and visual analogue scale (VAS) were calculated before and after operation. Segmental lordotic angle (SLA), lumbar lordotic angle (LLA) and segmental scoliosis angle (SSA), disc height (DH), posterior disc height (PDH), lateral disc height (LDH), foraminal height (FH), foramen width (FW) and foraminal cross-sectional area (FSCA) were measured before and after operation.

**Result:**

The VAS and ODI after operation were significantly improved as compared with those before operation. Compared with pre-operation, the DH, PHD increased by 67.6%, 94.6%, LDH increased by 107.4% (left), 101.7% (right), and FH increased by 30.2% (left), 34.5% (right). The FSCA increased by 93.1% (left), 89.0% (right), and the FW increased by 137.0% (left), 149.6% (right). The postoperative SSA was corrected by 74.5%, the postoperative SLA, LLA were corrected by 70.2%, 38.1%, respectively. All the imaging indexes were significantly improved (*p* < 0.01).

**Conclusion:**

The clinical efficacy and imaging data of OLIF in the treatment of LFS caused by low and moderate lumbar spondylolisthesis, intervertebral disc bulge and reduced intervertebral space height, degenerative lumbar scoliosis, articular process hyperplasia or dislocation have been well improved. OLIF may be one of the better surgical treatments for LFS caused by the above conditions.

## Introduction

Lumbar intervertebral foramen stenosis (LFS) is a common spinal degenerative disease. The nerve root in the narrow intervertebral foramen caused by various factors is compressed, which will show lower limb pain, numbness and weakness, intermittent claudication and other symptoms [1]. Although the pathology of LFS was first reported as early as 1927, LFS is often highly occult, and the imaging data are sometimes not obvious and easy to be ignored [2, 3]. At present, more attention is paid to the degeneration of lumbar intervertebral disc and central lumbar spinal canal stenosis, and there are few reports on LFS, so we often do not pay enough attention to LFS, which leads to missed diagnosis. it affects the judgment of the treatment plan, and even makes the choice of the operation plan unreasonable, resulting in poor postoperative effect and no relief of the symptoms of low back pain. Therefore, it is very important to correctly recognize, diagnose and treat LFS. There are a variety of factors leading to LFS, such as disc herniation, decrease of intervertebral foramen height, vertebral body slippage, isthmus spondylolisthesis, dislocation or hyperplasia of facet joint, thickening or fold of ligament, scar hyperplasia and so on. And with the advent of aging, degenerative spondylolisthesis, reduced intervertebral space height and scoliosis and other degenerative lumbar diseases with intervertebral foramen stenosis are also increasing.

At present, the surgical treatment of LFS is mainly for the enlargement of intervertebral foramen, including posterior lumbar decompression, intervertebral foramen endoscopy and lateral fusion [4]. Posterior lumbar decompression is mainly to remove the lamina and articular process and decompress the nerve under direct vision. Foraminal endoscope is to remove the compression of the exit nerve root, and sometimes it is necessary to partially remove the facet joint. The shortcomings and complications of posterior incision decompression and foraminoscopy include nerve injury, cerebrospinal fluid leakage, postoperative hematoma, destruction of articular process and so on [5]. At present, lateral fusion is considered to be a kind of indirect decompression, by implanting a large cage in the intervertebral space laterally, opening the intervertebral space, restoring the height of the intervertebral foramen, correcting the deformities in the coronal and sagittal position, and stretching the ligaments by cage, expand the neural pathway and relieve nerve compression [6, 7]. Retroperitoneal lateral fusion was first reported by Mayer et al. in 1997 [8], and OLIF was first reported by Silvestre et al [9] on this basis. This operation enters the extraperitoneal space through the muscle space of the abdominal external oblique muscle, the abdominal internal oblique muscle and the transverse abdominis muscle, and places the working channel in front of the psoas major muscle, which can be operated through the psoas major muscle and the great vascular space. Compared with the traditional posterior fusion, lateral lumbar interbody fusion has the advantages of less trauma, shorter operation time, less bleeding and less risk of nerve root and dural sac injury. In addition, the ligaments and intervertebral facet joints were preserved during OLIF, which did little damage to the posterior stable structure of the spine. And the implanted interbody fusion cage spans the whole width of the vertebral body, has good intervertebral stability, provides good intervertebral support, and can be used for better intervertebral foramen reduction and decompression. Previous studies have focused on the indirect decompression effect of OLIF in the treatment of lumbar spinal stenosis, but there is no special analysis of the clinical and imaging effects of OLIF in the treatment of LFS caused by different factors. This study retrospectively analyzed the patients with LFS caused by different factors, and used SeunghunLe’s LFS classification method to grade the lumbar intervertebral foramen stenosis [10]. The imaging data and clinical results before and after operation were analyzed, and the imaging and clinical effects of OLIF in patients with LFS caused by different factors were obtained.

## Materials and methods

The study was approved by the Ethics Committee of The Third Hospital of Shijiazhuang.

### General material

This study included 33 patients who received OLIF from January 2018 to May 2022, including LFS caused by mild to moderate lumbar spondylolisthesis, degenerative intervertebral disc disease, lumbar scoliosis, ligamentum flavum hypertrophy, articular process hyperplasia and dislocation. All patients were followed up for at least 12 months, and all patients underwent OLIF by the same operator. All patients had low back pain and / or lower limb pain that had no obvious effect after conservative treatment for more than 3–6 months. Patients with intervertebral foramen stenosis with central lumbar spinal stenosis need to have symptoms and evidence of intervertebral foramen stenosis. For patients with inaccurate preoperative diagnosis, the following signs or examinations are determined after combined with imaging examination: (1) Kemp sign is positive. (2) Selective nerve root closure to define the responsible segment. (3) DTI examination confirmed the compression of nerve root. Patients with combined posterior decompression, patients with fractures or patients with lateral fusion of infection, and patients with incomplete follow-up data are not included in the statistical scope. The general data of the patients before operation, including age, sex, body mass index(BMI) and so on, were recorded.

All 33 patients received OLIF combined with percutaneous pedicle screw fixation in 48 segments (intervertebral foramen stenosis grade Grade1-3). Of the 33 patients, 10 were male and 23 were female, with an average age of 61.9 years (33–83 years). There were 12 patients with LFS caused by lumbar spondylolisthesis (including true spondylolisthesis), 7 patients with LFS caused by degenerative scoliosis, 4 patients with LFS caused by hypertrophy of ligamentum flavum or hyperplasia of articular process. 10 patients with LFS caused by lumbar intervertebral disc degeneration and decrease of intervertebral space height. There were 52 intervertebral foramen in Grade 1, 33 intervertebral foramen in Grade 2 and 11 intervertebral foramen in Grade 3. There were 20 patients with single segment, 11 patients with 2 segments, and 2 patients with 3 segments (degenerative scoliosis with intervertebral foramen stenosis), including 5 patients with L2/3, 7 patients with L3/4, 29 patients with L4/5 and 7 patients with L5/S1. The above statistics did not include the intervertebral foramen of Grade0. All patients were implanted with 18–22 × 8–16 × 40-55 mm polyether ether ketone cage. Except for L5/S1 with 10°- 12° kyphosis fusion cage, 6°- 10° kyphosis was used, and allogeneic bone was filled in the fusion cage.

### Clinical outcomes

**Fig. 1:**
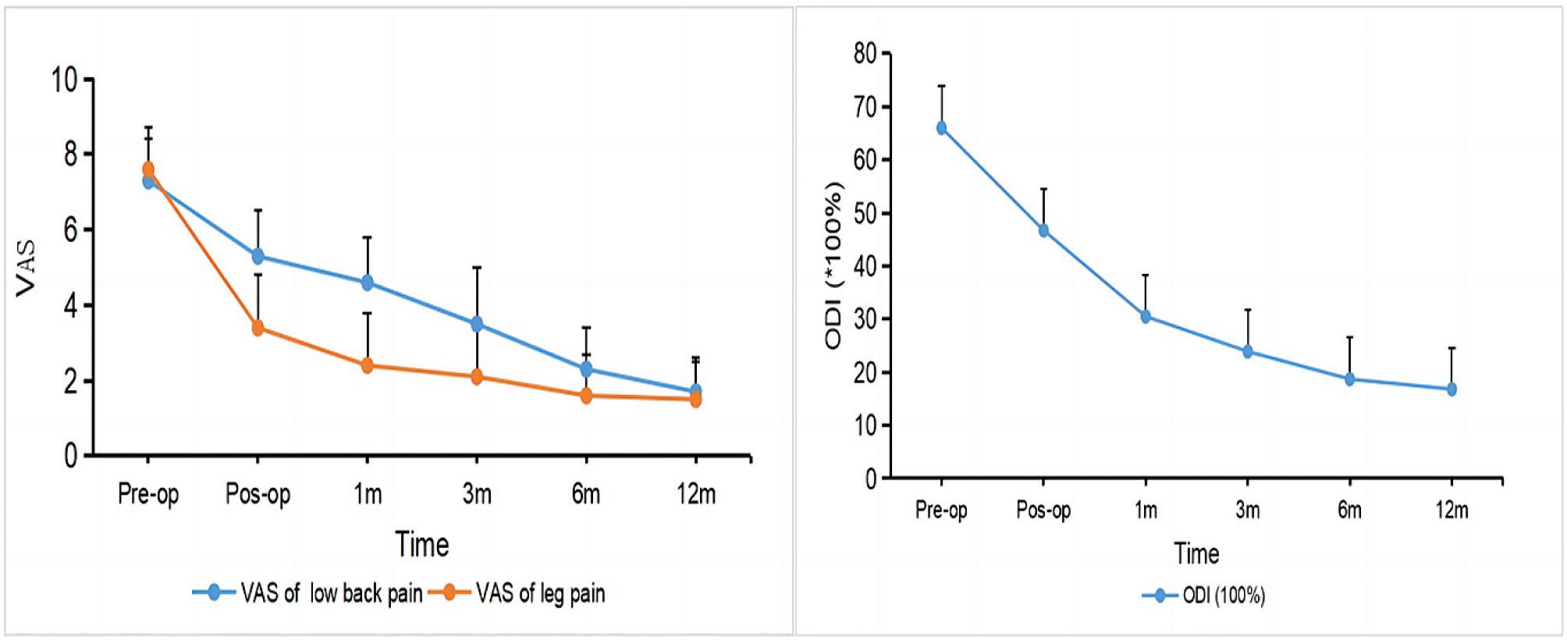
Postoperative visual analog scale of low back pain, visual analog scale of leg pain and Oswestry Disability Index were significantly reduced compared with those preoperative. VAS, visual analog scale; ODI, oswestry disability index

ODI was used to evaluate the lumbar function and daily activity of the patients before operation, immediately after operation (within 1 week), 1 month, 3 months, 6 months and 12 months. VAS score was used to evaluate the severity of low back pain and leg pain before, after and during follow-up (Fig. 1).

### Imaging assessments

**Fig. 2:**
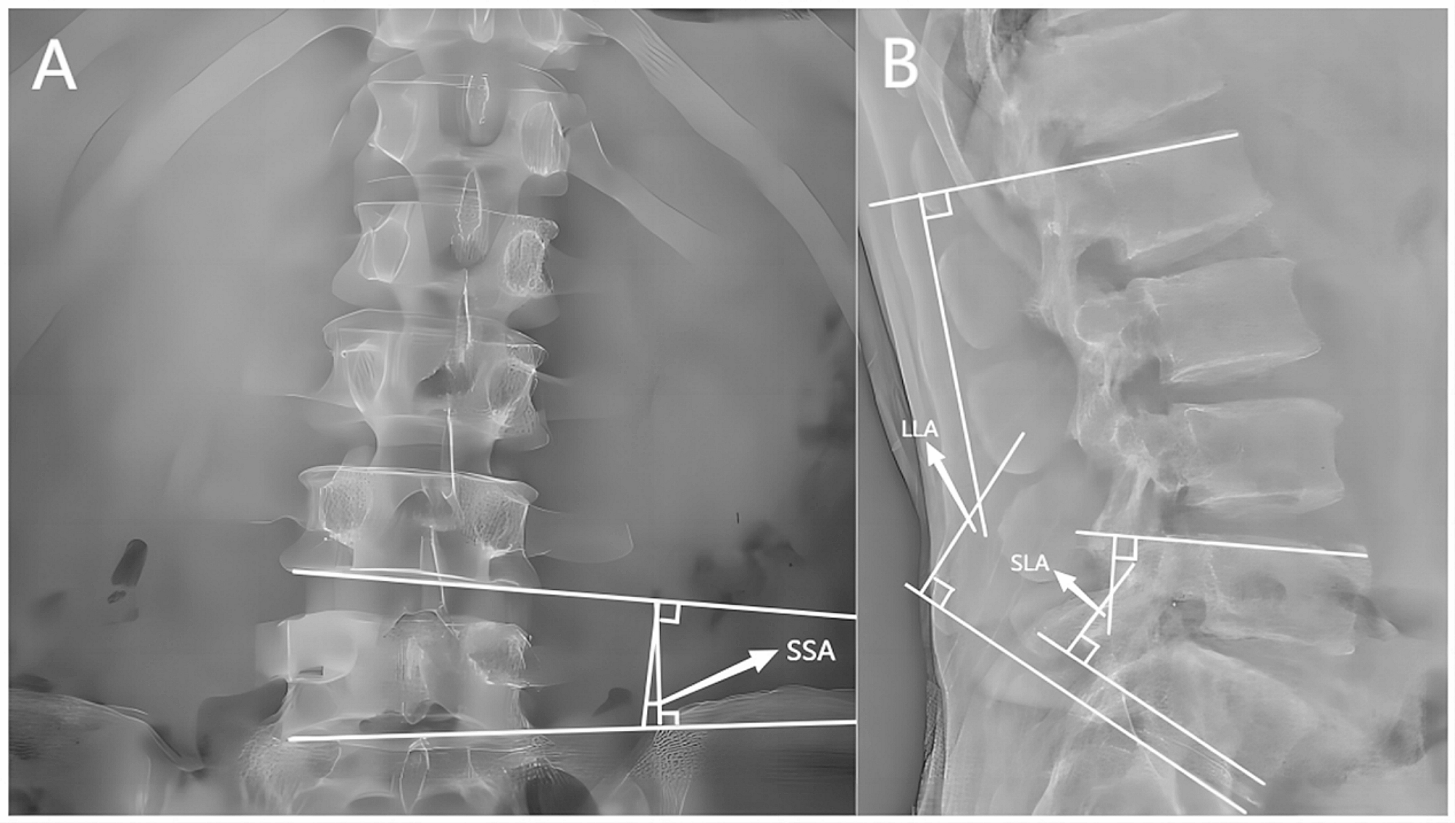
(**A**) The segmental scoliosis angle (SSA) is the cobb angle between the lower endplate of the superior vertebral body and the inferior endplate of the lower vertebral body on the coronal X-ray film of the surgical segment. (**B**) The segmental lordotic angle (SLA) is the cobb angle between the superior endplate of the superior vertebral body and the inferior endplate of the lower vertebral body on the sagittal X-ray of the lumbar. The lumbar lordotic angle (LLA) is the cobb angle between the superior endplate of L1 vertebral body and the superior endplate of S1 vertebral body in sagittal X-ray

**Fig. 3:**
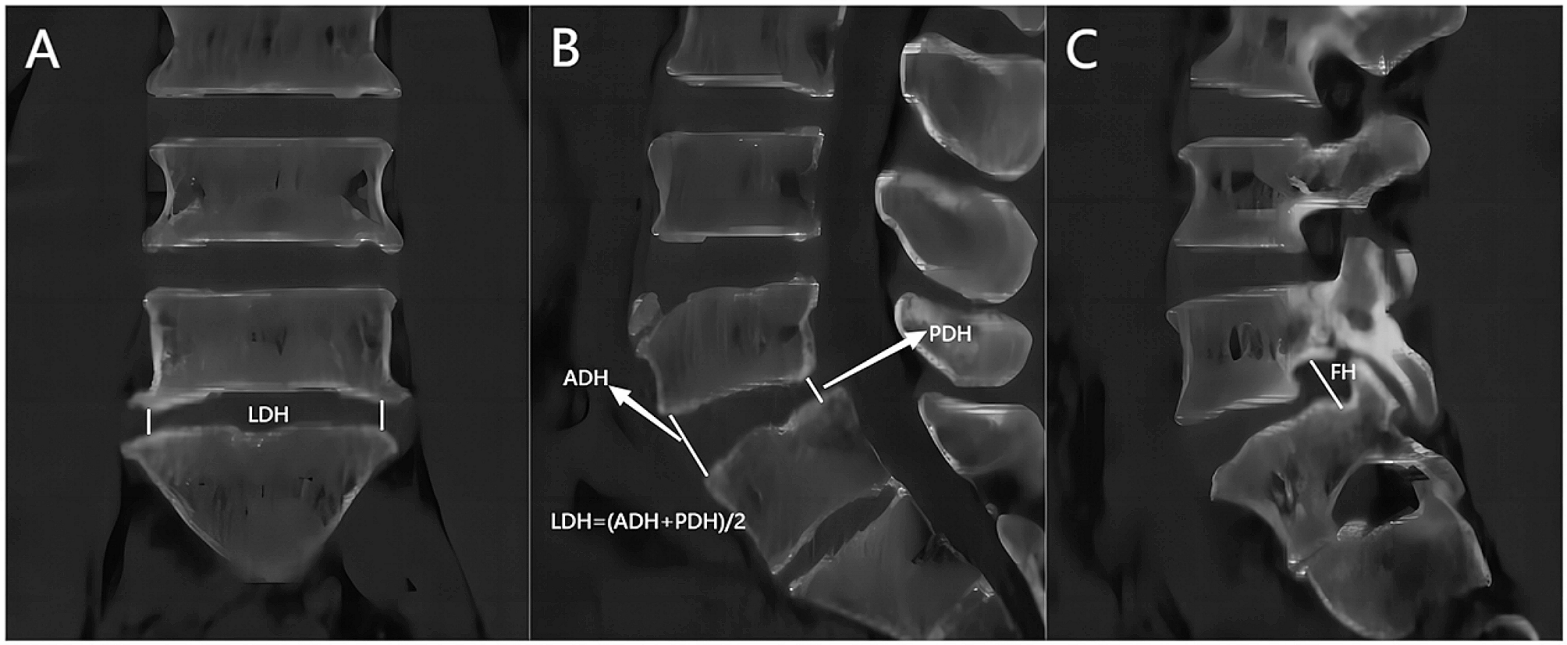
(**A**) The lateral disc height (LDH) was the lateral marginal height of midline intervertebral space of CT coronal vertebrae. (**B**) The disc height (DH) was the average of the disc height(ADH) and posterior disc height(PDH) in the sagittal CT. (**C**) The foraminal height (FH) was the distance between the inferior edge of the pedicle of the upper vertebral body of CT and the upper edge of the pedicle of the lower vertebral body

**Fig. 4:**
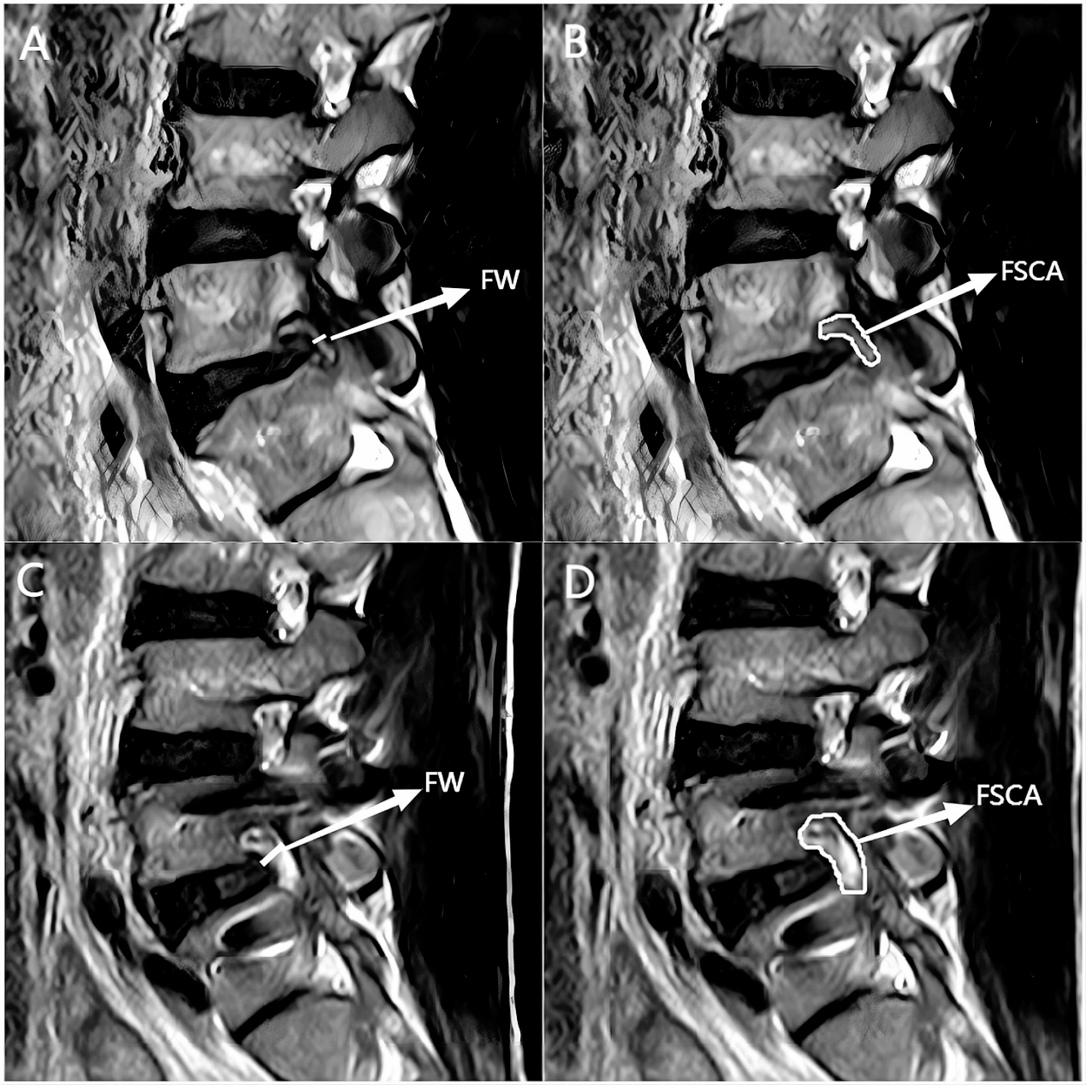
The foraminal cross-sectional area (FSCA) in the middle area of nerve foramen in T2-weighted MRI sagittal position was measured. The foramen width (FW) was the distance between the intervertebral disc in the middle region of the nerve foramen and the narrowest point of the posterior ligamentum flavum measured by T2-weighted MRI in sagittal position of the lumbar vertebra. **A** and **C** are the width of intervertebral foramen before and after operation, and **B** and **D** are the area of intervertebral foramen before and after operation

Lumbar standing X-ray, CT and MRI were performed before and immediately after operation (within 1 week). The segmental lordotic angle (SLA), lumbar lordotic angle (LLA) and segmental scoliosis angle (SSA) were measured before and immediately after operation. The SLA is the cobb angle between the superior endplate of the superior vertebral body and the inferior endplate of the lower vertebral body on the sagittal X-ray of the lumbar. The LLA is the cobb angle between the superior endplate of L1 vertebral body and the superior endplate of S1 vertebral body in sagittal X-ray. The SSA is the cobb angle between the lower endplate of the superior vertebral body and the inferior endplate of the lower vertebral body on the coronal X-ray film of the surgical segment (Fig. 2). The disc height (DH), posterior disc height (PDH), lateral disc height (LDH) and foraminal height (FH) were measured before and immediately after operation. The disc height (DH) was the average of the the disc height(ADH) and posterior disc height(PDH) in the sagittal CT. The lateral disc height (LDH) was the lateral marginal height of midline intervertebral space of CT coronal vertebrae. The foraminal height (FH) was the distance between the inferior edge of the pedicle of the upper vertebral body of CT and the upper edge of the pedicle of the lower vertebral body (Fig. 3). The foraminal cross-sectional area (FSCA) and foramen width (FW) were measured before and immediately after operation. The foraminal cross-sectional area (FSCA) in the middle area of nerve foramen in T2-weighted MRI sagittal position was measured. The foramen width (FW) was the distance between the intervertebral disc in the middle region of the nerve foramen and the narrowest point of the posterior ligamentum flavum measured by T2-weighted MRI in sagittal position of the lumbar vertebra (Fig. 4).

CT was performed 12 months after operation, and DH, PDH, LDH and FH were measured. Evaluation of intervertebral space fusion, evaluation criteria: the formation of continuous bone bridge in coronal or sagittal position of CT was judged as fusion. To evaluate the settlement of cage, the evaluation criteria: the 2 mm of the fusion cage exceeding the upper endplate of the lower vertebral body was judged as settlement.

### Statistical analysis

Continuous variables are expressed as mean ± standard deviation. Non-parametric data are reported as median (interquartile, IQR). Paired t test was used to compare the difference between preoperative and postoperative parameters. All statistical analyses were performed using SPSS 23.0 program. The statistically significant difference was considered to be *p* < 0.05.

## Results

### Baseline characteristics

The demographic characteristics of the patients are summarized in Table 1. A total of 33 patients (10 males and 23 females) were included in this study, with a total of 48 segments. Their average age was 61.9 years old (33–83 years), and the average body mass index (BMI) was 26.9 ± 3.7. The operative segments, the causes of intervertebral foramen stenosis and the grade of intervertebral foramen stenosis were statistically analyzed in Table 1.

**Table 1:** Baseline characteristics of 33 patients undergoing oblique lumbar interbody fusion for lumbar foramen stenosis

Characteristics	
Age (years), mean ± SD (range)	61.9 ± 11.3 (33–83)
Sex, *n* (%)	33
Male	10 (30.3%)
Female	23 (69.7%)
Body mass index (kg/m2), mean ± SD (range)	26.9 ± 3.7(21.1–37.5)
Causes of foraminal stenosis, *n* (%)	33
Spondylolisthesis (including true spondylolisthesis)	12 (36.4%)
Degenerative scoliosis	7 (21.2%)
Hypertrophy of ligamentum flavum or Hyperplasia of articular process	4 (12.1%)
Lumbar intervertebral disc degeneration and Decrease of intervertebral space height	10 (30.3%)
Level, *n* (%)	33
Single level	20 (60.6%)
Two level	11 (33.3%)
Three level	2 (6.1%)
Surgical level, *n* (%)	48
L2/3	5 (10.4%)
L3/4	7 (14.6%)
L4/5	29 (60.4%)
L5/1	7 (14.6%)
Foraminal stenosis grade, *n* (%)	96
Grade 1	52 (54.2%)
Grade 2	33 (34.4%)
Grade 3	11 (11.5%)

### Clinical outcomes

The low back pain VAS, leg pain VAS score and ODI index were significantly improved immediately after operation (within 1 week), 1 month, 3 months, 6 months and 12 months after operation. The VAS score of low back pain was significantly decreased from 7.3 ± 1.1 before operation to 1.7 ± 0.9 at 12 months after operation (*p* < 0. 01), representing an improvement of 77.7%. The VAS score of leg pain was significantly decreased from 7.6 ± 1.1 before operation to 1.5 ± 1.0 at 12 months after operation (*p* < 0. 01), representing an improvement of 79.8%. ODI was also significantly improved from 66.0 ± 10.8 before operation to 16.8 ± 2.1 at 12 months after operation (*p* < 0.01), representing an improvement of 74.2% (Table 2). There was no significant difference in postoperative low back pain VAS, leg pain VAS score and ODI index among the groups of LFS caused by various compression causes.

**Table 2:** Clinical outcomes

	Pre-op	Pos-op (within 1 week)	1month	3month	6month	12month	*P* (2-tailed)
VAS scores of low back pain	7.3 ± 1.1	5.3 ± 1.2	4.6 ± 1.2	3.5 ± 1.5	2.3 ± 1.1	1.7 ± 0.9	< 0.001
VAS scores of leg pain	7.6 ± 1.1	3.4 ± 1.4	2.4 ± 1.4	2.1 ± 1.3	1.6 ± 1.1	1.5 ± 1.0	< 0.001
ODI (*100%)	66.0 ± 10.8	46.7 ± 7.6	30.5 ± 6.5	23.9 ± 5.7	18.7 ± 4.0	16.8 ± 2.1	< 0.001

All the time periods after operation were statistically significant compared with those before operation. *P*-value < 0.05 is considered statistically significan. ODI : Oswestry Disability Index, VAS : Visual Analogue Scale

A total of 4 patients developed complications. One patient had retroperitoneal hematoma, which was found on MRI 3 days after operation. The patient had no special discomfort, delayed the time of getting out of bed, pressurized the abdominal belt, closely monitored the blood pressure and the number of red blood cells, and found hematoma absorption in the later stage of reexamination. Three patients developed numbness and weakness in front of the left thigh after operation, and the symptoms disappeared after 2 weeks of observation (Table 3).

**Table 3:** Postoperative complications, fusion rate and cage subsidence rate 12 months after surgery

Characteristics	
Totality	33
Fusion(12month), *n* (%)	31 (93.9%)
Cage subsidence(12month), *n* (%)	2 (6.1%)
Complications	--
Retroperitoneal hematoma, *n* (%)	1 (3.0%)
Numbness and weakness of left thigh	3 (9.1%)

### Imaging enaluation

DH, PDH, LDH, FH, the area of intervertebral foramen (FSCA) and FW were improved before and after operation. For SLA, LLA and SSA, the preoperative and postoperative measurement results showed that the improvement degree was different in LFS caused by different causes, and there was significant difference between preoperative and postoperative segmental scoliosis angle measurement results in LFS caused by scoliosis. For LFS caused by spondylolisthesis and intervertebral space stenosis, there were differences in SLA and LLA (Table 4). Overall, the postoperative DH increased by 67.6%, PDH increased by 94.6%, LDH increased by 107.4% (left) and 101.7% (right), and the FH increased by 30.2% (left) and 34.5% (right), respectively. The FSCA increased by 93.1% (left) and 89.0% (right), and the FW increased by 137.0% (left) and 149.6% (right), respectively. The postoperative SSA was corrected by 74.5%, the postoperative SLA and LLA were corrected by 70.2% and 38.1%, respectively. All the imaging indexes were significantly improved (all *p* < 0.01; Table 5).

**Table 4:** Comparison of decompression parameters in different causes of foraminal stenosis

	Spondylolisthesis (including true spondylolisthesis)	Degenerative scoliosis	Hypertrophy of ligamentum flavum or Hyperplasia of articular process	Lumbar intervertebral disc degeneration and Decrease of intervertebral space height
DH improvement rate (%); mean	54.7%	70.8%	85.2%	68.8%
PDH improvement rate (%); mean	62.7%	110.5%	147.9%	85.3%
LDH(left) improvement rate (%); mean	78.4%	123.4%	139.9%	102.7%
LDH(right) improvement rate (%); mean	94.8%	120.1%	113.6%	83.8%
FH(left) improvement rate (%); mean	20.9%	32.7%	61.0%	24.6%
FH(right) improvement rate (%); mean	28.6%	36.8%	47.2%	32.5%
FW(left) improvement rate (%); mean	77.5%	147.9%	183.8%	157.5%
FW(right) improvement rate (%); mean	172.9%	111.2%	155.4%	158.9%
FSCA(left) improvement rate (%); mean	75.1%	90.4%	155.5%	89.6%
FSCA(right) improvement rate (%); mean	74.2%	90.0%	84.7%	101.2%
SSA improvement rate (%); mean	69.3%	87.0%	92.4%	59.3%
SLA improvement rate (%); mean	45.8%	74.5%	42.9%	90.9%
LLA improvement rate (%); mean	38.8%	32.5%	29.9%	44.5%

DH: disc height; PDH: posterior disc height; LDH: lateral disc height; FH: foraminal height; FW: foramen width; FSCA: foraminal cross-sectional area; SSA: segmental scoliosis angle; SLA: segmental lordotic angle; LLA: lumbar lordotic angle

**Table 5:** Overall comparison of preoperative and postoperative imaging data

	Pre-op	Pos-op (within 1 week)	12month	P(2-tailed)	Improvement rate (%, pre-op vs. pos-op)
DH (mm)	7.4 ± 1.6	12.1 ± 1.7	10.7 ± 1.5	< 0.001	67.6%
PDH (mm)	5.3 ± 1.5	9.7 ± 2.2	8.6 ± 1.7	< 0.001	94.6%
LDH(left, mm)	5.9 ± 2.0	11.1 ± 1.6	9.9 ± 2.1	< 0.001	107.4%
LDH(right, mm)	6.0 ± 1.7	11.4 ± 2.0	10.0 ± 1.8	< 0.001	101.7%
FH(left, mm)	16.0 ± 3.2	20.1 ± 2.4	18.4 ± 2.1	< 0.001	30.2%
FH(right, mm)	15.8 ± 3.4	20.6 ± 3.1	18.5 ± 2.5	< 0.001	34.5%
FW(left, mm)	2.6 ± 1.2	5.3 ± 1.6	--	< 0.001	137.0%
FW(right, mm)	2.6 ± 1.2	5.5 ± 1.8	--	< 0.001	149.6%
FSCA(left, mm2)	64.5 ± 25.2	110.4 ± 30.8	--	< 0.001	93.1%
FSCA(right, mm2)	63.7 ± 23.5	110.3 ± 34.2	--	< 0.001	89.0%
SSA(degrees)	4.3 ± 4.1	0.9 ± 1.4	--	< 0.001	74.5%
SLA(degrees)	11.3 ± 5.1	16.9 ± 6.0	--	< 0.001	70.2%
LLA (degrees)	31.4 ± 7.2	42.3 ± 8.1	--	< 0.001	38.1%

Continuous variables were expressed as mean ± standard deviation (SD), *p*-value < 0.05 is considered statistically significan. DH: disc height; PDH: posterior disc height; LDH: lateral disc height; FH: foraminal height; FW: foramen width; FSCA: foraminal cross-sectional area; SSA: segmental scoliosis angle; SLA: segmental lordotic angle; LLA: lumbar lordotic angle

At 12 months after operation, the fusion rate was 93.9%. 2 patients did not meet the fusion standard, but the fusion cage did not shift obviously, and the clinical effect of the patients was good. There were 2 patients with cage subsidence, and the overall cage subsidence rate was 6.1% (Table 3). The clinical effect of the patients was good.

## Discussion

OLIF is different from the traditional posterior approach, lateral approach can fully clean the intervertebral space under direct vision, thoroughly remove the nucleus pulposus, remove the fibrous annulus to a large extent, scrape off most of the cartilage endplates, and provide an excellent fusion environment, which has a good fusion effect for patients with lumbar degeneration. Because the lateral approach clears out a large intervertebral space, a larger type of cage can be placed to stretch the intervertebral space, which can effectively restore the intervertebral space height and intervertebral foramen height, so that the ligamentum flavum folds are opened and the intervertebral disc is protruded back, so as to achieve the purpose of nerve decompression [5, 11]. Because the anterior longitudinal ligament, posterior longitudinal ligament and facet joints are preserved, multidirectional motion can be stabilized by the tension of the residual ring and ligament, and biomechanical analysis also suggests that larger cages can provide higher segmental stability [5, 12]. Related studies have shown that even for patients with severe stenosis of the central canal or intervertebral foramen with degenerative intervertebral disc disease, OLIF indirect decompression can still achieve good clinical results and radiological improvement [7, 13, 14]. Previous studies have focused on patients with lumbar spinal stenosis treated with OLIF or patients with lumbar spinal stenosis complicated with LFS, but there is no special study on the treatment of LFS with OLIF. This study mainly studies the patients with low back pain caused by different factors of LFS treated with OLIF.

LFS often has a high concealment, which is easy to be ignored in the diagnosis and treatment of daily diseases. The causes of LFS are diverse. With the advent of aging, there are more and more patients with LFS caused by degenerative intervertebral disc disease, such as reduced height of lumbar intervertebral space, intervertebral disc bulge, degenerative spondylolisthesis, scoliosis and so on. For LFS, the incidence of lower lumbar intervertebral disc stenosis is higher, first, the probability of lower lumbar intervertebral disc degeneration is higher, which is more likely to lead to lumbar instability and LFS, and because of anatomical characteristics, the nerve roots of the lower lumbar vertebrae are more sensitive to LFS caused by various factors [1]. Related studies have shown that because the shape of intervertebral foramen of L5/S1 is different from that of L1-4, L5/S1 intervertebral foramen stenosis is the most common [10, 15]. Hasegawa et al. believe that the reduction of posterior disc height to 4 mm or smaller or intervertebral foramen height to 15 mm or smaller is an important cause of nerve root compression [16].

At present, transforaminal lumbar interbody fusion (TLIF) and foraminal endoscopy are commonly used in the surgical treatment of lumbar intervertebral foramen stenosis. Although the anatomical structure of the posterior median approach of the lumbar spine is well known to clinicians, the exposure of the intervertebral foramen requires extensive dissection of paraspinal muscles, and destruction of facet joints, lamina isthmus and ligaments in order to fully decompress, thus affecting the stability of the spine and causing residual low back pain. For the lumbar posterior median approach, the decompression of the intervertebral foramen is easy to cause nerve root traction or inadequate decompression, which often leads to no remission or aggravation of postoperative neurological symptoms [17]. Especially for patients with bilateral intervertebral foramen stenosis, posterior lumbar decompression is more destructive to the posterior column of lumbar spine, and has a certain impact on lumbar stability and fusion rate. Compared with TLIF, OLIF does not need to destroy the posterior spinal tissue such as facet joint, does not destroy the stability of spine, has no nerve disturbance, has less nerve complications, and has less injury and less bleeding. Related studies also show that OLIF is superior to posterior approach in terms of operation time, blood loss, hospital stay and clinical effect [9, 18]. Foraminal endoscopic surgery is more suitable for patients with disc herniation or prolapse. Foramina endoscopy can not effectively decompress the LFS caused by lumbar spondylolisthesis, lumbar instability, scoliosis and obvious facet joint degeneration, let alone correct spondylolisthesis and scoliosis. In addition, it is necessary to use a ring saw to destroy part of the articular process when the intervertebral foramen is repaired by intervertebral foramen endoscopy. For patients with bilateral intervertebral foramen stenosis, OLIF has more advantages than intervertebral foramen endoscopy.

The lumbar intervertebral foramen is a typical dumbbell shape and is an axial channel [19]. Different from the spinal canal, the spinal canal is a sagittal channel, and the compression often comes from the front or rear. The principle of lateral approach decompression is that the intervertebral space is stretched and the intervertebral disc is reclaimed, and the ligament is opened, which is indirect decompression. The compression caused by LFS is often caused by the decrease of the height of the sagittal position, which leads to the narrowing of the superior and inferior diameter, or the narrowing of the anterior and posterior diameter due to the dislocation of the articular process, resulting in the decrease of the cross-sectional area of the sagittal plane. Therefore, we think that OLIF in the treatment of LFS can be called “reduction-decompression” of intervertebral foramen, and direct decompression of nerve root can be achieved by stretching reduction, which is more in line with the actual situation than indirect decompression. This method restores or improves the height, width and area of the intervertebral foramen, and restores the nerve root running channel, thus relieving the nerve compression, which is different from removing the articular process and from the ring saw in the foramen lens to repair the intervertebral foramen and allow to destroy part of the bone, but it can also be called a way of intervertebral foramen plasty. In this way, there is no nerve harassment, the incidence of postoperative nerve complications is low, and there is basically no risk of nerve injury. There is no need to remove the articular process, the bone is less destructive, and has no effect on the stability of the posterior column of the spine. In addition, OLIF can be used to treat LFS with a larger cage, which can ride over the epiphyseal ring, provide good intervertebral support, and perform better intervertebral foramen “reduction- decompression”. Moreover, it can effectively maintain the stability of the spine and intervertebral foramen [20], but it is necessary to pay attention to the failure of decompression caused by the settlement of the fusion cage, and the settlement of long-term results should be considered. We believe that posterior internal fixation can help to prevent the settlement of the fusion cage and avoid the failure of intervertebral foramen decompression to a great extent.

Compared with other lateral fusion techniques, such as extreme lateral interbody fusion(XLIF), OLIF can achieve the fusion of the whole lumbar spine of L1-S1, especially for L5/S1, which has a high incidence of intervertebral foramen stenosis, ATP-OLIF can perfectly solve the problem of high iliac spine. Different from ALIF operation, L5/S1OLIF can preserve the anterior longitudinal ligament, maintain better tension and improve the height of intervertebral space, so that the height of intervertebral foramen can be better improved, which is more beneficial to the decompression of intervertebral foramen. This study shows that for LFS caused by lumbar spondylolisthesis, scoliosis and decreased intervertebral space height, OLIF can not only correct lumbar spondylolisthesis and improve lumbar kyphosis, but also reduce and decompress bilateral lumbar intervertebral foramen. For unilateral intervertebral foramen stenosis caused by scoliosis, our studies have shown that OLIF can correct local segmental scoliosis, enlarge the concave intervertebral foramen, and has no effect on the convex intervertebral foramen. Related studies have also shown that OLIF has a good corrective effect on degenerative scoliosis [21]. For the LFS caused by severe calcification of ligamentum flavum and facet joint fusion, we think that distraction decompression is ineffective, it can not change the inherent compression and can not enlarge the bony structure. It is necessary to distinguish whether there is superior recess stenosis in the treatment of LFS. We think that the decompression effect of OLIF combined with lateral recess stenosis is not ideal. One study analyzed the radiological predictors of indirect decompression failure and concluded that bony lateral recess stenosis was a risk factor for lumbar indirect decompression failure [22]. Related studies have also shown that lateral fusion can not be solved for LFS caused by disc herniation, bone spur formation, severe facet arthropathy or synovial cyst [13].

### Limitation

This study is a single-center retrospective study, the sample size is small, the follow-up time is short, and further multicenter long-term large sample size analysis is needed in the later stage.

## Conclusion

This study shows that the clinical efficacy and imaging data of OLIF in the treatment of LFS caused by low and moderate lumbar spondylolisthesis, intervertebral disc bulge and intervertebral space height decrease, degenerative lumbar scoliosis, ligamentum flavum hypertrophy and articular process hyperplasia or dislocation have been well improved. It opened up the treatment of LFS and clarified the “reduction-decompression” principle of OLIF in the treatment of LFS. However, for the treatment of LFS with OLIF, it is necessary to identify the type of compression and identify whether there is bony lateral recess stenosis.

## Data Availability

No datasets were generated or analysed during the current study.
